# Comprehensive Proteomic Characterization of the Pectoralis Major at Three Chronological Ages in Beijing-You Chicken

**DOI:** 10.3389/fphys.2021.658711

**Published:** 2021-03-18

**Authors:** Jian Zhang, Jing Cao, Ailian Geng, Haihong Wang, Qin Chu, Linbing Yang, Zhixun Yan, Xiaoyue Zhang, Yao Zhang, Jie Dai, Huagui Liu

**Affiliations:** ^1^Institute of Animal Husbandry and Veterinary Medicine, Beijing Academy of Agriculture and Forestry Sciences, Beijing, China; ^2^Shanghai Bioprofile Technology Co., Ltd., Shanghai, China

**Keywords:** Beijing-You chicken, pectoralis major, chronological age, TMT-based quantitative proteomic analysis, parallel reaction monitoring

## Abstract

Chronological age is one of the important factors influencing muscle development and meat quality in chickens. To evaluate the protein expression profiles during skeletal muscle development, we performed a tandem mass tag (TMT)-based quantitative proteomic strategy in pectoralis major (breast muscle) of Beijing-You chicken (BYC) at the chronological age of 90, 120, and 150 days. Each chronological age contained 3 pooling samples or 15 birds (five birds per pooling sample). A total of 1,413 proteins were identified in chicken breast muscle with FDR < 1% and 197 of them were differentially expressed (fold change ≥1.2 or ≤0.83 and *p* < 0.05). There were 110 up- and 71 down-regulated proteins in 120 d vs 90 d group, 13 up- and 10 down-regulated proteins in 150 d vs 120 d group. The proteomic profiles of BYC at 120 d were very similar to those at 150 d and highly different from those at 90 d, suggesting that 120 d might be an important chronological age for BYC. Kyoto Encyclopedia of Genes and Genomes (KEGG) analyses indicated that these differentially expressed proteins were mainly involved in the pathway of glycolysis/gluconeogenesis, adrenergic signaling in cardiomyocytes, focal adhesion, oocyte meiosis and phagosome. Furthermore, some DEPs were quantified using parallel reaction monitoring (PRM) to validate the results from TMT analysis. In summary, these results provided some candidate protein-coding genes for further functional validation and contribute to a comprehensive understanding of muscle development and age-dependent meat quality regulation by proteins in chickens.

## Introduction

Muscle development and meat quality are traits that are comprehensively affected by age, gender, nutrition, and species (López et al., 2011; Díaz et al., 2012; Umaya, 2014; Tasoniero et al., 2018). Among these parameters, the animal’s chronological age is one of the most important factors that can strongly influence these traits (Peterson et al., 1959; Brant and Hanson, 1962; Mehaffey et al., 2006; Tougan et al., 2013). Moreover, the intramuscular fat (IMF) content also plays an important role in the flavor of chicken meat (Chizzolini et al., 1999; Ros-Freixedes et al., 2014; Li et al., 2019); in previous studies, the IMF content in chicken breast meat increased with age from 56 to 140 d, accompanied by enhanced flavor and taste of the meat (Liu et al., 2016; Cui et al., 2018).

The emerging quantitative proteomic technology allows the systematic study of static or perturbation-induced changes in proteome-wide expression profiles (Pan and Aebersold, 2007) and the discovery of different meat quality biomarkers (Paredi et al., 2012; Wu et al., 2015). Recently, several studies have been carried out to elucidate muscle development and meat quality traits in chickens through proteomics-based methods. For instance, Doherty et al. (2004) characterized the proteome of layer chicken breast muscle at specified time points from 1 to 27 d after hatching and 51 proteins were identified. In addition, Teltathum and Mekchay (2009) applied two-dimensional gel electrophoresis and matrix-assisted laser desorption ionization time-of-flight mass spectrometry to analyze proteomic changes in Thai indigenous chicken during the growth period. Five protein spots were characterized, and significant differences in energy metabolism and stress proteins were suggested to exist between age groups. In another work, Cai et al. (2018) characterized the whole muscle proteome and indicated that eight proteins were differentially expressed between normal and woody breast meat samples using two-dimensional gel electrophoresis. However, most of these studies were limited to gel-based proteomic approaches. In addition, early studies mainly focused on commercial broilers and layers, but an understanding of the molecular mechanisms underlying muscle development and meat quality due to Chinese indigenous chickens remains lacking.

The Beijing-You chicken (BYC), a famous Chinese native chicken breed, is more acceptable in China because of its taste, rich fragrance, and tenderness (Zhao et al., 2011). Liu et al. (2016) identified differentially expressed proteins in breast muscles of BYC at day 1 (hatching), 56 (fast growth age), 98 (marketing age), and 140 (first egg age). However, the criteria of identifying the marketing age of yellow chicken in China are varied due mainly to the customers’ preference, and the proteins profiles may exhibit great differences coming from the different chronological ages. Thus, we aim to assess the protein expression profiles for these different chronological ages of day 90 (fast growth age), 120 (extremely high quality marketing age), and 150 (first egg age for the bird population) of BYC. In this study, the tandem mass tag (TMT)-LC-MS/MS (Thompson et al., 2003) proteomic strategy was applied to systematically investigate the protein expression profiles, and related biomarkers contributing to breast muscle development and meat quality during these different chronological ages. In addition, some candidate proteins were validated by the targeted parallel reaction monitoring (PRM) method (Rauniyar, 2015; Ronsein et al., 2015). This study could improve our knowledge of the temporal expression profile during development and provide opportunities for exploring biological mechanisms and characterizing biomarkers underlying muscle development and meat quality.

## Materials and Methods

### Ethics Statement

All of the animal experiments were conducted in accordance with the guidelines for experimental animals established by the Ministry of Science and Technology (Beijing, China). Animal experiments were approved by the Science Research Department (in charge of animal welfare issues) of the Institute of Animal Husbandry and Veterinary Medicine, Beijing Academy of Agriculture and Forestry Sciences (Beijing, China) and the approval number was BAAFS-IAHVM20191009.

### Animals and Tissue Sampling

Ninety one-day-old female BYC birds were obtained from the Institute of Animal Husbandry and Veterinary Medicine, Beijing Academy of Agriculture and Forestry Sciences and the individual birds had the same genetic background. All the birds entered the experiment at the same time and were randomly distributed into three replicate groups; each group comprised 30 BYC birds. Birds were raised in an environmentally controlled room with three floor pens under the recommended environmental and nutritional conditions for BYC. Feed and water were provided ad libitum during the experiment.

For each chronological age (90, 120, or 150 d), five birds of similar weight from each replication were weighed individually to obtain the live weight before the birds were electrically stunned and killed by exsanguination, and each chronological age contained 15 birds. Breast muscle from both sides was used. After 10 cube samples (200 mg per cube) of each left filet were snap-frozen in liquid nitrogen and stored at −80°C until the TMT and PRM test, the remaining meat samples from the left side were used to obtain the IMF content. The right filets were collected as well and were weighed and stored at 4°C for measurement of meat quality characteristics.

### Meat Quality Characteristics

Drip loss was measured as described previously (Berri et al., 2008). The Warner-Bratzler shear force (WBSF) analysis was performed as described previously (Geesink et al., 1995). Shear force determinations were conducted on a TMS-PRO (FTC Co., United States) equipped with a WBSF head at a crosshead speed of 200 mm/min. The shear force on the breast meat sample was represented as the arithmetic mean value of six cuts. The filet samples, which were individually vacuum sealed in cook bags, were stored at 4°C for 24 h and then cooked in a water bath at 85°C until the internal temperature of cooked samples reached 80°C. The internal temperature of meat samples and water bath were monitored with thermometer (Reed SD-947, Reed Instruments, Canada). Cooked samples were chilled to room temperature, drained of liquid and patted dry. The samples were further cut into strips with the size of 1.0 cm (width) × 0.5 cm (thickness) × 2.5 cm (length). The strips parallel to the muscle fiber were prepared from the medial portion of the filet and sheared vertically. The IMF content of breast muscle was determined by extraction with petroleum ether in a Soxhlet apparatus (Zerehdaran et al., 2004) and expressed as a percentage of the dry weight of the muscle.

### Protein Extraction

An appropriate amount (100 mg) of chicken breast muscle from each sample was ground with liquid nitrogen. Then, 1.5 mL of lysis buffer (4% SDS, 100 mM DTT, 150 mM Tris-HCl, pH 8.0) was added to the ground meat for protein extraction. Samples were disrupted, ultrasonicated, and then boiled for 3 min. Then, after centrifugation (16,000 × *g*, 15 min, 4°C), the supernatant was collected, and the protein content was quantified using a BCA Protein Assay Kit (Bio-Rad, United States). In order to minimize the difference due to subject-to-subject variation and better identifies characteristics of the population, the pooling strategy was carried out in this study (Kendziorski et al., 2005). In brief, every five samples from each stage were pooled using equal amounts of protein, followed by diluting nine pools (three pools per age) to the same concentration with Tris-buffered saline (TBS) before protein digestion. Each pool was tested twice.

### Protein Digestion

A total of 300 μg of protein from each diluting pool sample, which was made up of five individual samples, was taken for protein digestion according to the FASP procedure (Wiśniewski et al., 2009). Briefly, the detergent (i.e., SDS), DTT and other low-molecular-weight components were removed using 200 μL of urea (UA) buffer (8 M UA, 150 mM Tris-HCl, pH 8.0) by repeated ultrafiltration (Microcon units, 30 kDa) facilitated by centrifugation. Then, iodoacetamide in UA buffer (final concentration 50 mM) was added to block reduced cysteine residues, followed by incubation of the samples for 20 min in darkness. The filter was washed with 100 μL of UA buffer three times and then with 100 μL of 40 mM NH_4_HCO_3_ twice. Finally, the protein suspension was digested with 4 μg of LysC/trypsin (Promega) in 40 μL of 40 mM NH_4_HCO_3_ overnight at 37°C for 18 h, followed by termination of the digestion procedure with an appropriate amount of formic acid (FA). The resulting peptides were collected by centrifugation. Then, the peptides were desalted using a C18 cartridge (Sigma-Aldrich) and resolved with OD280 peptide quantification.

### TMT Labeling and Peptide Fractionation

Peptides (100 μg) were labeled with TMT reagents according to the manufacturer’s instructions (Thermo Fisher Scientific). Briefly, after the sample was dissolved in 100 μL of 50 mM triethyl ammonium bicarbonate (TEAB) solution at pH 8.5, the TMT reagent was dissolved in 41 μL of anhydrous acetonitrile. The peptide mixture was incubated at room temperature for 1 h. Then, 8 μL of 5% hydroxylamine was added into the sample, and the mixture incubated for 15 min to quench the reaction. The multiplex labeled samples were pooled together in equal amounts, followed by lyophilization. The High-pH Reversed-Phase Peptide Fractionation Kit (Thermo Fisher Scientific) was used for fractionation of dried TMT-labeled peptides. Eventually, the sample was collected and pooled into 15 fractions. The peptides of each fraction were dried and reconstituted with 0.1% FA for LC-MS analysis.

### LC-MS/MS Analysis

The fractionated peptides were subjected to LC-MS/MS analysis and analyzed on a Q Exactive Plus mass spectrometer coupled to an Easy nLC 1200 (Thermo Fisher Scientific). Peptides from each fraction were loaded onto a C18 reversed-phase column (15 cm long, 75 μm ID, 3 μm) in buffer A (0.1% FA) and separated with a linear gradient of buffer B (85% acetonitrile and 0.1% FA) at a flow rate of 300 nL/min over 75 min. The gradient was as follows: 0–5 min, linear gradient from 2 to 5% buffer B; 5–50 min, linear gradient from 5 to 23% buffer B; 50–60 min, linear gradient from 23 to 40% buffer B; 60–65 min, linear gradient from 40 to 100% buffer B; 65–75 min, buffer B maintained at 100%. MS data were acquired using a data-dependent top-20 method, dynamically choosing the most abundant precursor ions from the survey scan (300–1,800 m/z) for MS/MS acquisition. Determination of the target value was based on predictive automatic gain control (pAGC). The AGC target value was 3.0 × 10^6^ and the maximum injection time was 50 ms for full MS, and the target AGC value was 1.0 × 10^5^ and the maximum injection time was 100 ms for MS2. The dynamic exclusion duration was 30 s. Survey scans were acquired at a resolution of 70,000 at m/z 200, and the resolution for MS/MS was set to 17,500 at m/z 200. The normalized collision energy was 30. The instrument was run with peptide recognition mode enabled. The mass spectrometry proteomics data have been deposited to the ProteomeXchange Consortium via the PRIDE (Perez-Riverol et al., 2019) partner repository with the dataset identifier PXD023871.

### Database Search

The resulting LC-MS/MS raw files were imported into MaxQuant software (version 1.6.1.0) for data interpretation and protein identification against the database UniProt-gallus gallus-35124-20190830.fasta (released in August 2019 and including 35,124 sequences), which was sourced from the protein database at https://www.uniprot.org/uniprot/?query=taxonomy:9031. An initial search was performed with a precursor mass window of 6 ppm. The search followed an enzymatic cleavage rule of trypsin/P and allowed two maximal missed cleavage sites and a mass tolerance of 20 ppm for fragment ions. The modification set was as follows: fixed modification: carbamidomethyl (C), TMT6plex (K), and TMT6plex (*N*-term); variable modification: oxidation (M) and acetyl (protein *N*-term). A minimum of six amino acids was required for peptides, and ≥1 unique peptide was required per protein. For peptide and protein identification, the false discovery rate (FDR) was set to 1%. Normalized TMT reporter ion intensity was used for peptide and protein quantification. The relative quantitative protein analysis of samples was conducted using the MaxQuant algorithms^1^.

### Data Statistics and Bioinformatics Analysis

Analyses of bioinformatics data were carried out with Perseus software, Microsoft Excel and R statistical computing software. Significant DEPs were screened with a fold change (FC) ratio cutoff of >1.20 or <0.83 and *P*-values < 0.05 among two pairwise comparison groups (120 d vs 90 d and 150 d vs 120 d). Expression data were grouped together by hierarchical clustering according to the protein level. To annotate the sequences, information was extracted from UniProtKB/Swiss-Prot, Kyoto Encyclopedia of Genes and Genomes (KEGG), and Gene Ontology (GO). GO and KEGG enrichment analyses were carried out with Fisher’s exact test, and FDR correction for multiple testing was also performed. GO terms were grouped into three categories: biological process (BP), molecular function (MF), and cellular component (CC). Enriched GO and KEGG pathways were nominally statistically significant at the *P* < 0.05 level. Construction of protein–protein interaction (PPI) networks was also conducted by using the STRING database with Cytoscape software.

### Targeted Protein Quantification by LC-PRM/MS Analysis

To further validate the protein expression level gain through TMT quantification, additional quantification through LC-PRM/MS analysis was performed. PRM analysis was performed on a Q Exactive HF-X mass spectrometer (Thermo Fisher Scientific). Methods optimized for charge state and retention times for the most significantly regulated peptides were generated experimentally using 1 to 3 unique peptides of high intensity and confidence for each target protein. Briefly, the TMT protocol was used for peptide preparation. Then, tryptic peptides were loaded on C18 stage tips for desalting prior to reversed-phase chromatography on an Easy nLC-1200 system (Thermo Fisher Scientific) with a constant flow rate of 300 nL/min and the following liquid gradient: from 0 to 5 min, mobile phase B (0.1% FA in 85% acetonitrile) was increased from 2 to 5%; from 5 to 45 min, mobile phase B was increased from 5 to 23%; from 45 to 50 min, mobile phase B was increased from 23 to 40%; from 50 to 52 min, mobile phase B was increased from 40 to 100%; and from 52 to 60 min, mobile phase B was held at 100%. The mass spectrometer was operated in positive ion mode with the following parameters: The full MS1 scan was acquired with a resolution of 60,000 (at m/z 200), an AGC target value of 3.0 × 10^6^, and a maximum ion injection time of 250 ms. Full MS scans were followed by 20 PRM scans at a resolution of 30,000 (at m/z 200) with an AGC value of 1.0 × 10^6^ and a maximum injection time of 200 ms. The targeted peptides were isolated with a 1.6Th window and fragmented at a normalized collision energy of 28 in a higher-energy collisional dissociation (HCD) cell. The raw data were analyzed using Skyline 4.1 (MacCoss Lab, University of Washington) to obtain the signal intensities of individual peptide sequences.

For the PRM-MS data, each sample’s average base peak intensity was extracted from the full scan acquisition using RawMeat (version 2.1, VAST Scientific^2^). The normalization factor for sample *N* was calculated as *f*_*N*_ = the average base peak intensity of sample *N*/the median of the average base peak intensities of all samples. The area under the curve (AUC) for each transition from sample *N* was multiplied by this factor. After normalization, the AUC of each transition was summed to obtain AUCs at the peptide level. The relative protein abundance was defined as the intensity of a certain peptide.

### Statistical Analysis

The parameters are shown as averages and standard errors. Data on carcass and meat characteristics were obtained and compared in a completely randomized design by using the General Linear Model procedure of SAS (version 9.2, SAS Institute Inc., Cary, NC, United States). Differences among ages were separated by Duncan’s multiple range tests. Significant differences were based on *P* < 0.05.

## Results

### Growth Characteristics and Meat Quality

The breast muscle samples for the three chronological ages were tested to characterize the following traits: live weight, IMF content, WBSF value and drip loss (Table 1). The live weight at 90 d (945 g) was lower (*P* < 0.05) than those at 120 and 150 d (1,205 and 1,295 g), which did not differ from each other (*P* > 0.05). As expected, the WBSF value and IMF content showed a significant increasing trend with the age of the chickens (*P* < 0.05). The WBSF value and IMF content at 90 d (18.52 N, 2.44%) were significantly lower (*P* < 0.05) than those at 120 d (29.15 N, 4.68%) and 150 d (30.60 N, 5.66%), which exhibited no significant difference (*P* > 0.05). Moreover, the drip loss exhibited the lowest value at 120 d (*P* < 0.05). These results indicated that there was a rapid increase in live weight during the stage from 90 to 120 d, and there was a larger amount of IMF deposition at this stage than there was from 120 to 150 d. This finding suggested that bird development reaches a relatively moderate level after a rapid increase in growth rate and IMF accumulation, suggesting that the stage from 90 to 120 d might be an important chronological stage for BYC.

**TABLE 1:** Characteristics of breast meat of Beijing-You chickens at different ages (mean ± SD, *n* = 15).

Item	90 d	120 d	150 d
Live Weight (g)	945 ± 87^b^	1205 ± 136^a^	1295 ± 136^a^
Drip Loss (%)	3.90 ± 1.08^a^	2.83 ± 0.78^b^	3.61 ± 1.11^a^
IMF (%)	2.44 ± 2.25^b^	4.68 ± 2.88^a^	5.66 ± 2.72^a^
WBSF(N)	18.52 ± 4.84^b^	29.15 ± 8.46^a^	30.62 ± 6.90^a^

*^*a,b*^Means within the same row followed by a different superscript are significantly different (*P* ≤ 0.05). IMF, intramuscular fat; WBSF, Warner-Bratzler shear force.*

### Protein Identification and Quantification

**FIGURE 1:**
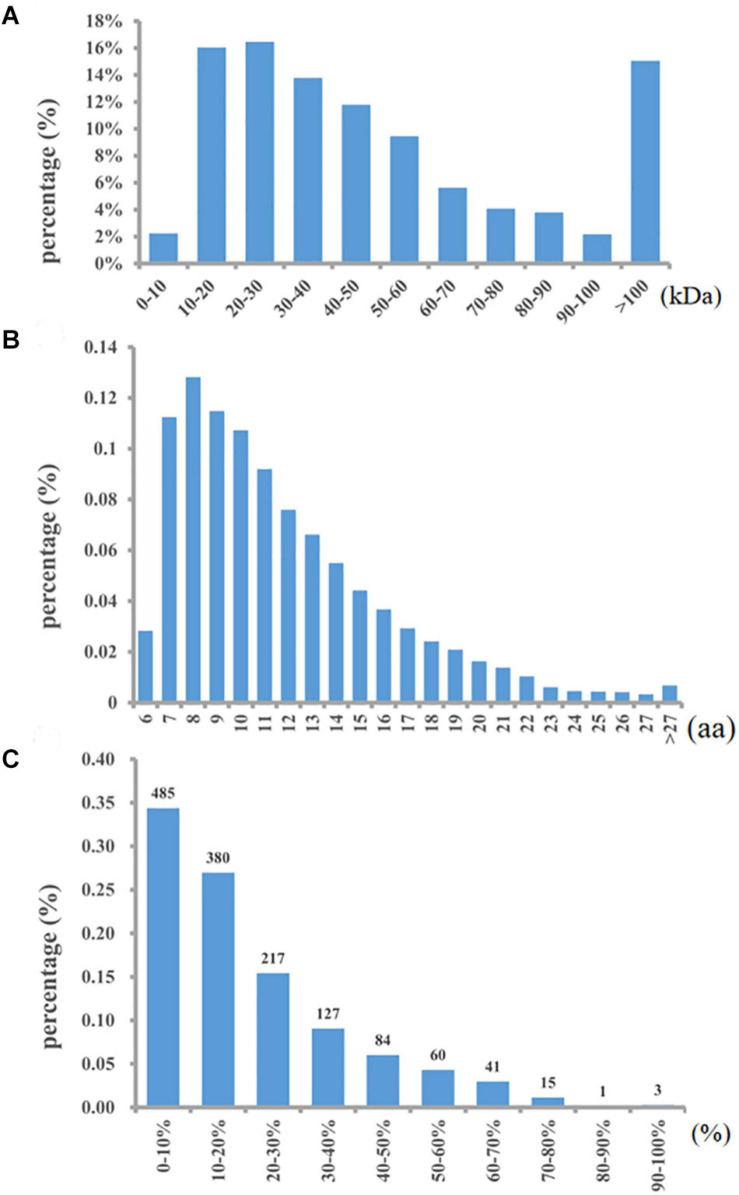
Distributions of all identified proteins. Mass **(A)**, length **(B)**, and coverage **(C)**.

A total of 10,661 peptides and 1,413 proteins were identified with an FDR <1%. Details of all the accurately identified proteins are shown in Supplementary Table S1. Among all the identified proteins, 57.89% had a molecular weight of approximately 10-50 kDa, and 15% were more than 100 kDa in weight (Figure 1A). Approximately 49.02% of the peptides were 6–10 amino acids in length (Figure 1B). In addition, more than 82% of the proteins included at least two unique peptides. The proteins were identified with low sequence coverage; approximately 76.57% of proteins had less than 30% sequence coverage (Figure 1C).

### DEPs of Comparison Groups

**FIGURE 2:**
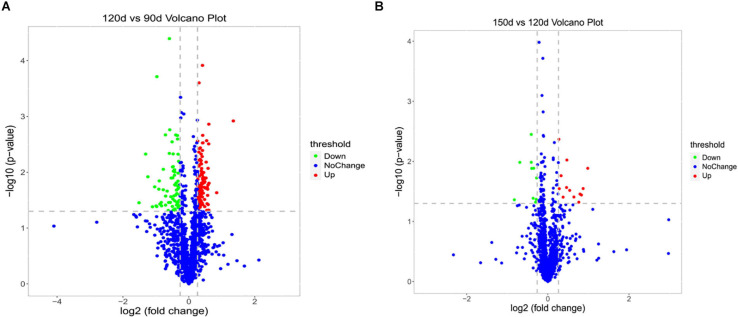
Volcano plots of the univariate statistical analysis results. 120 d vs 90 d **(A)** and 150 d vs 120 d **(B)**. The red dots indicate significantly upregulated proteins (*P* < 0.05 and FC > 1.2), and the green dots indicate significantly downregulated proteins (*P* < 0.05 and FC < 0.83). The blue dots represent proteins with non-significant (*P* > 0.05 or 0.83 < FC < 1.2) differences in expression.

To assess dynamic changes in proteins at different chronological ages, a total of 197 DEPs (after deredundancy analysis) were identified (FC > 1.2 or <0.83 and *P* < 0.05) from the two pairwise comparison groups (120 d vs 90 d and 150 d vs 120 d) (Supplementary Table S2). In the 120 d vs 90 d comparison group, 181 proteins (110 upregulated and 71 downregulated) were significantly changed. Moreover, 23 DEPs (13 upregulated and 10 downregulated) were significantly changed in the 150 d vs 120 d comparison group (Supplementary Table S2). The number of DEPs in the 120 d vs 90 d comparison group was larger than that in the 150 d vs 120 d comparison group, indicating that there were much more changes in the protein profiles from 90 to 120 d than from 120 to 150 d. Two volcano plots were generated to visualize the data for the two pairwise comparison groups (120 d vs 90 d and 150 d vs 120 d) (Figures 2A,B).

Hierarchical cluster analysis was performed to better visualize the differences in protein abundance among pairwise comparison groups, and the results were visualized as a heat map. As illustrated in Supplementary Figure S1, DEPs at three different chronological ages (90, 120, and 150 d) were identified. Samples from chickens of these ages displayed different color distributions; however, the three biological replicates of each sample group exhibited similar color layouts. These results confirmed that there were characteristic differences in the proteome among samples of different chronological ages. When the Euclidean distance was increased, the samples from 120 to 150 d were aggregated into a cluster and separated from the 90 d samples, which corroborated the difference in phenotypic traits. All of these results indicated that these differentially abundant proteins were the source of the variation in response to increasing chronological age.

### Functional Analysis of DEPs From 90 to 120 d

**FIGURE 3:**
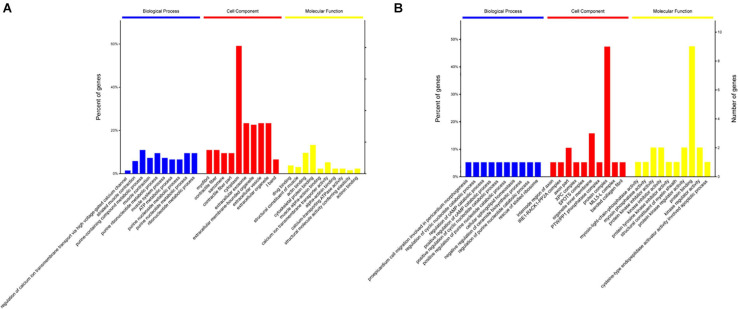
Go enrichment analysis of differentially expressed proteins in 120 d vs 90 d **(A)** and 150 d vs 90 d **(B)**.

**FIGURE 4:**
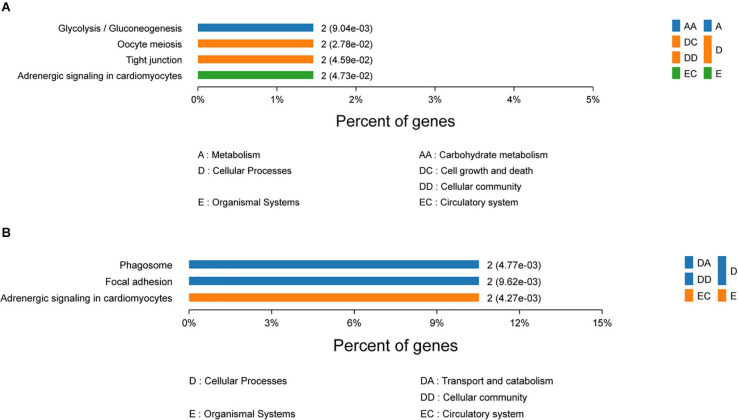
KEGG pathway enrichment analysis of differentially expressed proteins in 120 d vs 90 d **(A)** and 150 d vs 120 d **(B)**.

To gain insights into the functions of DEPs in the 120 d vs 90 d comparison group, GO and KEGG enrichment analyses were carried out to identify the meaningful biological functions of the DEPs. GO annotation of 181 DEPs identified 1,671, 296, and 379 categories in the BP, CC, and MF categories, respectively (Supplementary Table S3), of which 334, 105, and 99 categories were significant (*P* < 0.05) by Fisher’s exact test. In these pairwise comparisons, the DEPs in the BP group were mainly distributed in muscle system process, glycosyl compound metabolic process, cofactor metabolic process, generation of precursor metabolites and energy organophosphate metabolic process. The DEPs in CC group were mainly enriched in the terms cytoplasm, extracellular exosome, extracellular membrane-bounded organelle, vesicle and myosin complex. Additionally, the DEPs in the MF group were mainly distributed in cytoskeletal protein binding, heme-copper terminal oxidase activity, oxidoreductase activity, ligase activity and peptide transporter activity. The top 10 categories in BP, CC, and MF are shown in Figure 3A. The glycolysis/gluconeogenesis, oocyte meiosis, tight junction, and adrenergic signaling in cardiomyocyte pathways were significantly enriched by KEGG pathway enrichment analysis in the 120 d vs 90 d comparison group (Figure 4A and Supplementary Table S4), and more than half of these pathways are involved in energy metabolism and muscle development.

**FIGURE 5:**
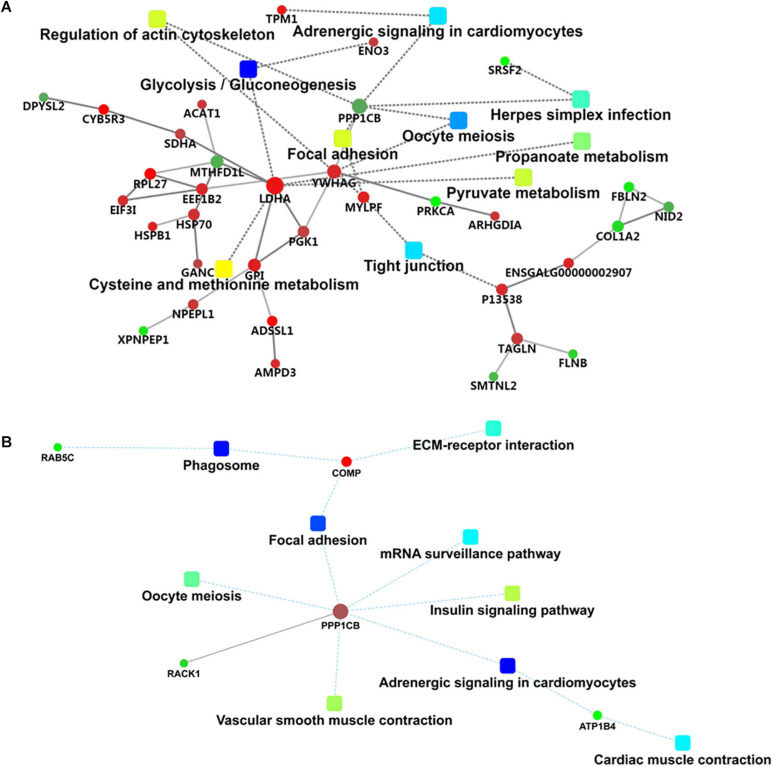
Interaction network of differentially expressed proteins in 120 d vs 90 d **(A)** and 150 d vs 120 d **(B)**.

Protein–protein interaction network analysis was performed to show the interaction networks of some important DEPs in the comparison groups. The PPI network analysis focused on several key pathways of energy metabolism and muscle development from 90 to 120 d (Figure 5A). The results showed that L-lactate dehydrogenase A chain (LDHA), beta-enolase (ENO3), and fructose-bisphosphate aldolase (ALDOC) were enriched in the glycolysis/gluconeogenesis pathway, while myosin regulatory light chain 2 (MYLPF) and myosin heavy chain (P13538) were derived from the tight junction pathway and tropomyosin alpha-1 chain (TPM1) was derived from the adrenergic signaling in cardiomyocytes pathway. Striking increases were observed for LDHA, ENO3, ALDOC, MYLPF, P13538 and TPM1 from 90 to 120 d (Supplementary Table S4), which was closely associated with muscle growth through the glycolysis/gluconeogenesis, tight junction and adrenergic signaling in cardiomyocytes pathways.

### Functional Analysis of DEPs From 120 to 150 d

For the 150 d vs 120 d comparison group, GO annotation of 23 DEPs identified 758, 170, and 117 categories in the BP, CC and MF categories, respectively (Supplementary Table S3), of which 189, 66, and 46 categories were significant (*P* < 0.05) by Fisher’s exact test. In this pairwise comparison, the DEPs in the BP group were mainly distributed in positive regulation of mitochondrial depolarization, embryonic morphogenesis, myoblast proliferation, negative regulation of response to oxidative stress and generation of precursor metabolites and energy. Regarding the CC group, the DEPs were mainly located in the IRE1-RACK1-PP2A complex, axon part, organelle inner membrane, fibrillar collagen trimer, and extrinsic component of mitochondrial inner membrane. Additionally, the DEPs in the MF group were mainly distributed in kinase regulator activity, enzyme binding, cysteine-type endopeptidase regulator activity involved in apoptotic process, enzyme inhibitor activity and channel inhibitor activity. The top 10 categories in BP, CC, and MF are shown in Figure 3B. The adrenergic signaling in cardiomyocytes, phagosome and focal adhesion pathways were significantly enriched by KEGG pathway enrichment analysis in the 150 d vs 120 d comparison group (Figure 4B, Supplementary Table S4).

For the PPI network in this stage (Figure 5B), the serine/threonine-protein phosphatase PP1-beta catalytic subunit (PPP1CB), cartilage oligomeric matrix protein (COMP), ras-related protein Rab-5C (RAB5C), protein ATP1B4 (ATP1B4), and receptor of activated protein C kinase 1 (RACK1) proteins were involved in some important pathways, including adrenergic signaling in cardiomyocytes, focal adhesion, phagosome, the insulin signaling pathway, insulin resistance, vascular smooth muscle contraction, oocyte meiosis, and extracellular matrix (ECM)–receptor interaction.

### Protein Validation by PRM

To assess the validity of the TMT data, PRM was used to examine the relative levels of ten important functional proteins at different chronological ages. As shown in Table 2, the protein expression levels obtained by TMT were confirmed by quantifying the expression levels of some proteins through PRM-MS analysis. Five proteins (LDHA, ENO3, MYBPC2, Q91348, and UCH-L1) related to muscle development, two proteins (CACNA2D1 and FKBP12.6) related to muscle contraction, and three proteins (COL1A2, GSTAL2, and HSP70) related to meat quality were selected for PRM analysis. The PRM results had good correlation with the TMT data (Table 2). The Pearson correlation coefficient between the TMT and PRM results was *R*^2^ = 0.897, indicating that these quantitative results were strongly convincing.

**TABLE 2:** Comparison of the quantification results between TMT and PRM analyses of the 10 candidate proteins.

Accession	Gene Name	Protein	Group Ratio (TMT)^a^			Group Comparison (PRM)^b^		
			120 d/90 d	150 d/120 d	150 d/90 d	120 d/90 d	150 d/120 d	150 d/90 d
P00340	LDHA	L-lactate dehydrogenase A chain	1.41	1.05	1.48	1.40	1.05	1.47
P07322	ENO3	Beta-enolase	1.24	0.97	1.20	1.17	1.21	1.41
A0A3Q3ATJ1	CACNA2D1	VWFA domain-containing protein	1.20	0.94	1.12	1.55	0.89	1.39
A1IMF0	UCH-L1	Ubiquitin carboxyl-terminal hydrolase	1.44	0.95	1.36	1.77	0.86	1.52
P16419	MYBPC2	Myosin-binding protein C, fast-type	1.24	0.96	1.20	1.35	0.96	1.28
Q8QGU2	FKBP12.6	Peptidylprolyl isomerase	0.77	0.88	0.68	0.83	0.90	0.75
P02467	COL1A2	Collagen alpha-2(I) chain	0.58	1.73	1.01	0.38	2.35	0.90
Q91348	Q91348	6-Phosphofructo-2-kinase/fructose-2,6-bisphosphatase	1.04	1.24	1.29	1.03	1.50	1.54
A0A0A0MQ61	GSTAL2	Uncharacterized protein	1.46	1.25	1.82	1.28	1.37	1.76
Q7SX63	HSP70	Heat shock protein 70	1.28	0.99	1.27	1.24	1.01	1.26

*^*a*^Fold changes in protein abundance from the comparisons among 90, 120, and 150 d by tandem mass tag (TMT).*

*^*b*^Fold changes in protein abundance from the comparisons among 90, 120, and 150 d by parallel reaction monitoring (PRM).*

## Discussion

Proteomic analysis was known as a powerful technique for studying the protein expression patterns, and has been widely carried out in identifying proteome changes of skeletal muscle at different development stages in chickens (Doherty et al., 2004; Teltathum and Mekchay, 2009; Liu et al., 2016; Ouyang et al., 2017). For example, Ouyang et al. (2017) characterized the proteome of breast muscle during embryonic development. Protein expression profiles were also investigated in the breast muscle of BYC at ages 1, 56, 98, and 140 days and Thai indigenous chickens at 0, 3, 6, and 18 weeks of age (Teltathum and Mekchay, 2009; Liu et al., 2016). In this study, we performed a TMT-LC-MS/MS-based proteomic analysis of breast muscle of BYC at 90, 120, and 150 days of age, and a total of 197 DEPs (after deredundancy analysis) were identified (FC > 1.2 or <0.83 and *P* < 0.05) from the two pairwise comparison groups (120 d vs 90 d and 150 d vs 120 d). These DEPs were mainly associated with glycolysis/gluconeogenesis, ECM–receptor interaction, focal adhesion, calcium signaling pathway, cysteine and methionine metabolism, pyruvate metabolism, oocyte meiosis, oxytocin signaling pathway and cardiac muscle contraction. The results of this study could strengthen our knowledge of the proteins temporal expression profile and make a complementary to previous findings. Some discussion on the key proteins of each chronological age, and their association with meat quality were provided as below.

### Muscle Development

Fontanesi et al. (2012) reported that LDHA associated with average daily gain in Italian Large White pigs. Qiu et al. (2010) mapped LDHA gene close to myogenic differentiation 1 (MyoD1), which is involved in the regulation of energy metabolism and protein transport processes, and suggested that LDHA gene could have great effect on muscle development. Wu et al. (2008) performed the genomic structure, polymorphism, expression of ENO3 and showed that ENO3 might affect not only skeletal muscle growth during muscle development but also meat flavor and carcass quality. All these studies demonstrated that glycolysis/gluconeogenesis, cardiac muscle contraction and dilated cardiomyopathy (DCM) might be the related pathways involved in muscle development (Liu et al., 2016). In the present study, we found the L-lactate dehydrogenase A chain (LDHA), and Beta-enolase (ENO3) were lower expressed in 90 d than those in 120 and 150 d, which did not differ from each other (Supplementary Table S2). In addition, Our data showed that the weight of 90 d (945 g) was lower (*P* < 0.05) than 120 d and 150 d (1,205 and 1,295 g), which did not differ from each other (*P* > 0.05). The results showed that the grow speed of breast muscle from 90 to 120 d is about threefold mort than the stage from 120 to 150 d (Table 1). These results suggested that the proteins, such as LDHA and ENO3, might have important effect on breast muscle development in BYC.

### IMF and Collagen Deposition

The deposition of IMF had extremely closely relationship with the developmental stages (Chartrin et al., 2007; Liu et al., 2016). Previous studies demonstrated that ECM–receptor interaction might form a network with pathways related to lipid metabolism to influence the deposition of IMF (Cui et al., 2012; Zhang et al., 2019). Zhang et al. (2019) identified 2,039 DEGs through a pairwise comparison of preadipocytes at different stages of differentiation and reported that pathways related to ECM–receptor interaction, focal adhesion pathway and PPAR signaling pathway were significantly enriched for IMF-derived preadipocyte differentiation. Li et al. (2019) performed RNA-Seq analysis of breast muscle from Gushi chicken at two physiological stages and showed that differentially expressed mRNAs and IncRNAs were mainly involved in ECM–receptor interaction, glycerophospholipid metabolism. Collagen is an abundant connective tissue protein and is contributing factor to variation in meat tenderness and texture (Uitto and Larjava, 1991). Collagen cross-linking increased with age was the major contributor to meat generally tougher from older birds (Fletcher, 2002; Rajkumar et al., 2016).

In the present study, we found that the expression of type I collagen alpha 2 (COL1A2) was lower in 120 d than those in 90 and 150 d, which did not differ from each other (Supplementary Table S2). Furthermore, pathway enrichment analysis show the proteins, including serine/threonine-protein phosphatase PP1-beta catalytic subunit (PPP1CB) and COMP (A0A3Q2UFV6) were also involved in the ECM–receptor interaction and focal adhesion (Supplementary Table S4). Among these proteins, PPP1CB, a novel adipogenic activator, has been reported to played an important function in regulating meat quality, which mainly associated with focal adhesion, cAMP signaling pathway, cGMP-PKG signaling pathway, adrenergic signaling in cardiomyocytes and oxytocin signaling pathway. Moreover, PPP1CB was significantly lower at 120 d compared to 90 and 150 d, which exhibiting the same pattern as the COL1A2. This result was consistent with the data published by Cho et al. (2015), who indicated that PPP1CB is essential for adipocyte differentiation, which supplied that PPP1CB might play a prominent role in regulating meat quality traits, such as juiciness and tenderness due to adipogenic process. COL1A2 was suggested through transcriptome sequencing to affect the growth of Jinghai yellow chickens recently (Wu et al., 2020). In addition, our result showed that IMF and WBSF exhibited an increasing trend with the increase in physiological stages (*P* < 0.05). The IFM and WBSF of 90 d (2.44%, 18.52 N) was lower (*P* < 0.05) than 120 d (4.68%, 29.15 N) and 150 d (5.66% and 30.60 N), respectively, which did not differ from each other (*P* > 0.05) (Table 1). These results were consistent with the results of previous studies suggesting that proteins involved in collagen and lipid metabolism were associated with tenderness and accumulation of IMF during chicken development (Li et al., 2010, 2019; Zhang et al., 2019). Hence, we speculated that the proteins, such as COL1A2, PPP1CB, and COMP having an important relationship to the ECM-receptor and focal adhesion pathway, played a critical role in the lipid metabolism and the meat tenderness due to fat and collagen accumulated with aging. These data were complementary to the findings reported by Liu et al. (2016) who identified pathways involved in muscle development that were dominant during the rapid muscle growth period (from 56 to 98 d) and suggested apolipoprotein A-1 (APOA1) and Heat shock protein beta 1 (HSPB1) as molecular markers for IMF deposition in chickens.

### Water-Holding Capacity

ATP2A1 encodes sarco/endoplasmic reticulum Ca^2+^-ATPase (SERCA), which is a pump that transports calcium ions from the cytoplasm into the sarcoplasmic reticulum (SR) (Periasamy and Kalyanasundaram, 2007). SERCA and the calcium release channel were demonstrated to be the most important regulators of the intracellular Ca^2+^ concentration (Xing et al., 2017), which included the mRNA and protein expression of SERCA1 and αRYR in the pectoralis major muscles of broilers from the normal and pale, soft and exudative (PSE) groups, and the expression was found to be significantly decreased in the PSE group compared to the normal group. In this study, we found ATPase Ca^2+^ transporting gene 1 (ATP2A1), calcium-transporting ATPase (ATP2B4), ryanodine receptor gene 3 (RYR3), tropomyosin alpha-1 chain (TPM1), and calcium channel and voltage-dependent alpha-2/delta subunit 1 (CACNA2D1) were lower expressed in 90 d than those in 120 d (Supplementary Table S2), which was accompanied by the decrease in the drip loss (from 3.90 to 2.83%) (Table 1). Moreover, pathway analysis showed that these proteins were involved in the calcium signaling pathway, the oxytocin signaling pathway, cardiac muscle contraction, and adrenergic signaling in cardiomyocytes. These results were consistent with the results of previous studies, supporting the views that proteins involved in calcium channel moderation and muscle contraction were associated with water-holding capacity (WHC) (Paião et al., 2013; Yalcin et al., 2019). Notably, heat shock protein 70 (HSP70) was more abundant at 120 d than at 90 d (Supplementary Table S2). Similar results have been reported in previous studies (Di Luca et al., 2011, 2013), which found that the upregulated expression of HSP70 could prevent protein denaturation and lead to reduced drip loss. One possible explanation could be that these proteins play an essential role in the maintenance of cellular integrity in response to stress (Wu et al., 2015). In addition, it is of interest to point out that there were no significant differences in proteins expression of ATP2A1, ATP2B4, RYR3, TPM1, CACNA2D1, and HSP70 between 120 and 150 d; however, significant differences of drip loss (from 2.83 to 3.61%) were noted between the stages. Taken together, our data collected in the present study provided the evidence that drip loss in pectoralis major muscles might be influenced by various proteins, and the relative affection of these proteins may be changing with the different physiological stages.

### Sexual Maturation

Notably, RACK1, a multifaceted scaffolding protein, was less abundant at 150 d than at 120 d (Supplementary Table S2), and this protein functions as a hub for spatiotemporal orchestration of signaling events across diverse pathways (Adams et al., 2011). Kadrmas et al. (2007) found RACK1-specific enrichment in the ovary and demonstrated that RACK1 is essential at multiple steps of Drosophila development, particularly in oogenesis. These results suggested that RACK1 might play a key role in sexual maturation in local BYC as well, which reached sexual maturity at the age of 150 d.

In conclusion, the present study examined the differences in protein expression levels in chicken breast muscle for three different chronological ages through TMT-LC-MS/MS-based quantitative proteomic analysis. The temporal expression patterns of some age-dependent proteins were determined and eluciated the dynamic changes in the chicken breast muscle proteomes with aging as well. Overall, the present work could strengthen our view of the temporal expression profile during development and identify novel biomarkers for genetic breeding of chickens.

## Data Availability Statement

The datasets presented in this study can be found in online repositories. The names of the repository/repositories and accession number(s) can be found below: ProteomeXchange Consortium via the PRIDE partner repository and PXD023871.

## Ethics Statement

The animal study was reviewed and approved by Science Research Department of the Institute of Animal Husbandry and Veterinary Medicine, Beijing Academy of Agriculture and Forestry Sciences (Beijing, China).

## Author Contributions

JZ: performed the experiments, analyzed the data, and wrote the manuscript. JC and AG: collected the samples. HW and QC: project administration. ZY, XZ, and YZ: performed the experiments. LY and JD: analyzed the data. HL: designed the study and reviewed the manuscript. All authors have read and approved the final manuscript.

## Conflict of Interest

LY and JD were employed at Shanghai Bioprofile Technology Co., Ltd., Shanghai, China. The remaining authors declare that the research was conducted in the absence of any commercial or financial relationships that could be construed as a potential conflict of interest.

## Footnotes

1 http://www.maxquant.org

2 www.vastscientific.com

## Supplementary Material

Supplementary Figure 1 Heatmap analysis of all differentially expressed proteins in three different chronological ages of breast muscle.

Supplementary Table 1 The identified 10,661 peptides and 1,413 proteins list in chicken breast muscle by TMT.

Supplementary Table 2 Differentially expressed proteins for 120 d vs 90 d, 150 d vs 120 d, and 150 d vs 90 d.

Supplementary Table 3 Gene Ontology (GO) analysis of 1,413 proteins in chicken breast muscle.

Supplementary Table 4 KEGG pathway enrichment analysis in chicken breast muscle.
