# Supplementary figures and images for: Comprehensive Proteomic Characterization of the Pectoralis Major at Three Chronological Ages in Beijing-You Chicken
Source: Front Physiol. 2021 Mar 18;12:658711. doi: 10.3389/fphys.2021.658711 (PMC8012914; doi:10.3389/fphys.2021.658711)

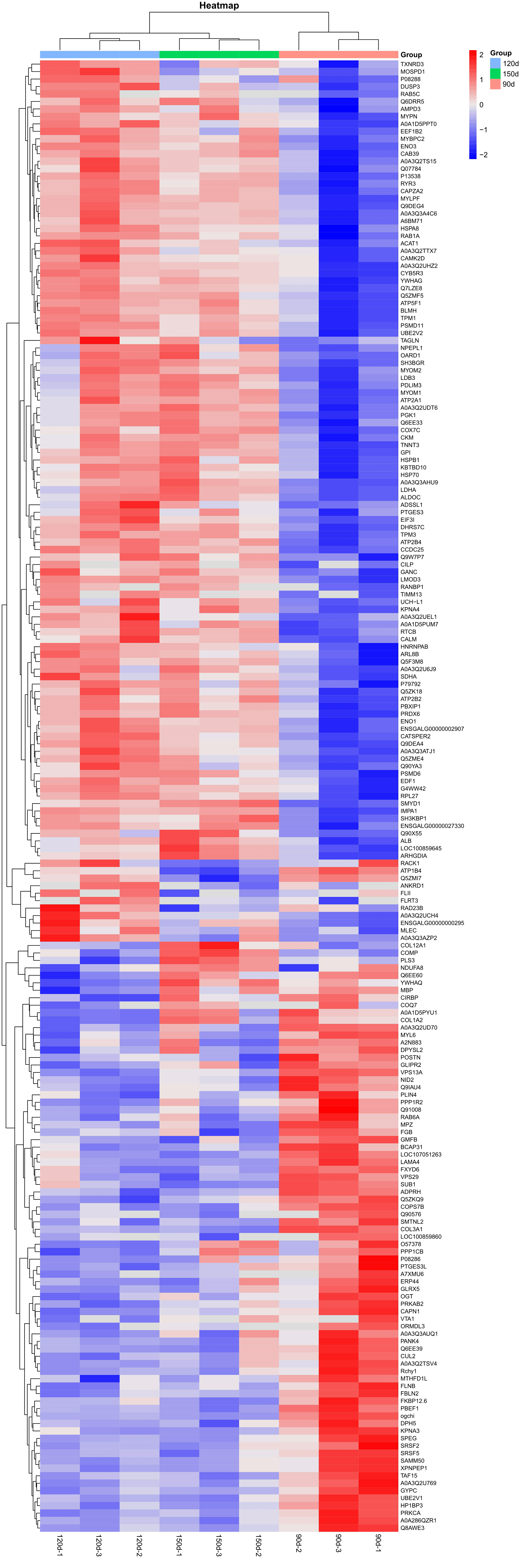

Supplement: Supplementary Figure 1 — Heatmap analysis of all differentially expressed proteins in three different chronological ages of breast muscle. [file Image_1.png]
